# Few-Shot Transfer Learning for Diabetes Risk Prediction Across Global Populations

**DOI:** 10.3390/medicina62010007

**Published:** 2025-12-19

**Authors:** Shrinit Babel, Sunit Babel, John Hodgson, Enrico Camporesi

**Affiliations:** 1 Morsani College of Medicine, University of South Florida, Tampa, FL 33602, USA; 2 Judy Genshaft Honors College, University of South Florida, Tampa, FL 33620, USA; sunitbabel@usf.edu; 3 Department of Anesthesiology and Perioperative Medicine, University of South Florida, Tampa, FL 33620, USA; john_hodgson@teamhealth.com (J.H.); ecampore@usf.edu (E.C.); 4 TEAMHealth Anesthesia, Tampa General Hospital, Tampa, FL 33606, USA

**Keywords:** domain adaptation, Type 2 diabetes mellitus, few-shot learning, global health, transfer learning

## Abstract

*Background and Objectives*: Type 2 diabetes mellitus (T2DM) affects over 537 million adults worldwide and disproportionately burdens low- and middle-income countries, where diagnostic resources are limited. Predictive models trained in one population often fail to generalize across regions due to shifts in feature distributions and measurement practices, hindering scalable screening efforts. *Materials and Methods*: We evaluated a few-shot domain adaptation framework using a simple multilayer perceptron with four shared clinical features (age, body mass index, mean arterial pressure, and plasma glucose) across three adult cohorts: Bangladesh (n = 5288), Iraq (n = 662), and the Pima Indian dataset (n = 768). For each of the six source-target pairs, we pre-trained on the source cohort and then fine-tuned on 1, 5, 10, and 20% of the labeled target examples, reserving the remaining for testing; a final 20% few-shot version was compared with threshold tuning. Discrimination and calibration performance metrics were used before and after adaptation. SHAP explainability analyses quantified shifts in feature importance and decision thresholds. *Results*: Several source → target transfers produced zero true positives under the strict source-only baseline at a fixed 0.5 decision threshold (e.g., Bangladesh → Pima F_1_ = 0.00, 0/268 diabetics detected). Few-shot fine-tuning restored non-zero recall in all such cases, with F_1_ improvements up to +0.63 and precision–recall gains in every zero-baseline transfer. In directions with moderate baseline performance (e.g., Bangladesh → Iraq, Iraq → Pima, Pima → Iraq), 20% few-shot adaptation with threshold tuning improved AUROC by +0.01 to +0.14 and accuracy by +4 to +17 percentage points while reducing Brier scores by up to 0.14 and ECE by approximately 30–80% (suggesting improved calibration). All but one transfer (Iraq → Bangladesh) demonstrated statistically significant improvement by McNemar’s test (*p* < 0.001). SHAP analyses revealed population-specific threshold shifts: glucose inflection points ranged from ~120 mg/dL in Pima to ~150 mg/dL in Iraq, and the importance of BMI rose in Pima-targeted adaptations. *Conclusions*: Leveraging as few as 5–20% of local labels, few-shot domain adaptation enhances cross-population T2DM risk prediction using only routinely available features. This scalable, interpretable approach can democratize preventive screening in diverse, resource-constrained settings.

## 1. Introduction

Type 2 Diabetes Mellitus (T2DM) is a progressive metabolic disorder and among the most pressing global health challenges, increasing in incidence and prevalence in both high-income and low- and middle-income countries (LMICs). The International Diabetes Federation suggests that the global prevalence of diabetes was estimated at 537 million in 2021, and is projected to rise to 643 million by 2030, with T2DM making up nearly 90–95% of all cases [1,2]. Its presentation, management, and data availability vary across countries and regions because of differences in cultural and dietary habits, socioeconomic factors, and local healthcare infrastructure [3].

Early, noninvasive risk stratification is a critical aspect in tackling the global rise in T2DM. Despite long-established diagnostic and screening guidelines, the prevalence of undiagnosed T2DM is also noteworthy, especially in LMICs, where healthcare infrastructure and screening capacity are limited [4]. Indeed, lab testing may remain the gold standard for screening and diagnosis; however, predictive models that utilize routinely available characteristics and demographic features can offer a scalable alternative in settings where fundamental measures, such as fasting glucose assays, blood pressure techniques, and anthropometric norms, may not generalize appropriately [1,5]. A reliable model that can flag high-risk individuals for confirmatory testing would spare many from unnecessary blood draws while initiating timely lifestyle modifications for others.

Machine learning has been widely applied in identifying patients at high risk for diabetes [6]. Several past studies have developed models trained on the PIMA Indian dataset, showing strong predictive performance with only a few easily collectible patient attributes. Although they perform well within the contexts in which they were trained, the models’ generalizability to other cohorts is questionable [7,8]. Subtle differences in dietary patterns, hypertension treatment practices, or data entry conventions can falter ML-based risk stratification [9]. Furthermore, collecting newer data in resource-constrained settings may not be feasible [10]. In many global health datasets, especially those focused on non-communicable diseases such as diabetes, data availability is sparse, and more importantly, the labeling is inconsistent [11,12]. Only a small number of shared clinical features may be available across cohorts, and the clinical relevance of these features can change depending on how they are measured and recorded. Therefore, a model trained on a dataset from one region or population may fail to generalize to another if the underlying associations between predictors and disease outcomes differ.

Transfer learning, specifically domain adaptation, allows models trained on one “source” cohort to adapt to another population, the target domain, using only a small number of samples [13]. It assumes that while the distribution of input features may shift across cohorts, the underlying task remains constant. Techniques such as feature transformation, reweighting, or adversarial alignment can be used to minimize the divergence between the source and target domains [13]. Transfer learning is widely used in medical image and speech analysis, although its utility remains underexplored in precision medicine or clinical prediction with tabular health data [14]. For example, a recent effort to bridge this gap is TabuLa-8B (2024), a large language model adapted for tabular data prediction, which shows strong performance on unseen datasets with minimal training samples [15].

Few-shot fine-tuning represents one such framework for adapting models to new settings. A small fraction of labeled examples from the target domain is used to fine-tune and adjust a source model, such as by re-weighting the contributions of individual shared features [15,16]. In clinical scenarios where acquiring large, labeled datasets for generating a new model is not feasible, few-shot learning can be a powerful approach to improve model generalization without the overhead of model redevelopment. Med-BERT (2021) and EHRSHOT (2023) are two recent examples that use pre-trained models to adapt to large-scale electronic health records [17].

That being said, transfer learning remains underexplored in the context of global health, especially for risk prediction tasks using clinical data. In heterogeneous and low-resource settings, cross-population transfer is especially relevant where data sparsity and measurement inconsistencies could otherwise jeopardize model reliability [18]. Tools that can learn from minimal local data while leveraging broader trends from other populations can help democratize predictive modeling in global health. In this study, we assess the few-shot domain adaptation for T2DM risk modeling across culturally and geographically distinct cohorts, and explore how interpretability tools can illuminate commonalities and unique drivers of diabetes risk in each setting. We hypothesize that a simple multilayer perceptron will generalize more reliably across global contexts using few-shot fine-tuning.

## 2. Methods

**Figure 1:**
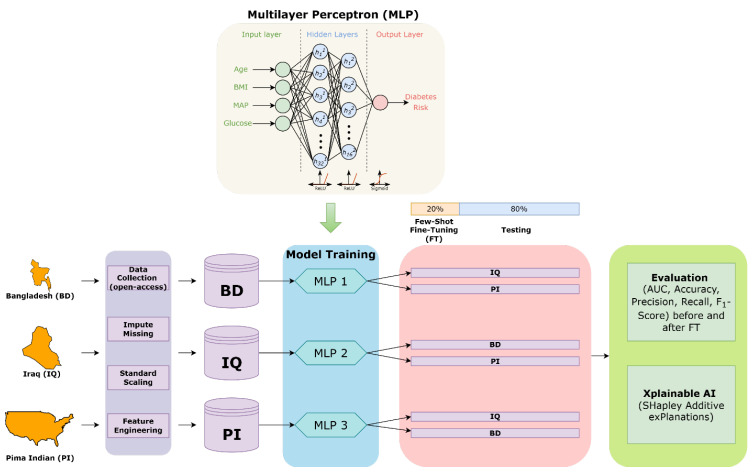
Transfer Learning and Research Methodology. Three open-access cohorts from Bangladesh (BD), Iraq (IQ), and the Pima Indian population (PI) were preprocessed to include four standardized clinical features: age, body mass index (BMI), mean arterial pressure (MAP), and blood glucose. A multilayer perceptron (MLP) model with two hidden layers was trained separately on each source dataset. MLP was ideal for our use given its flexibility for fine-tuning under few-shot conditions, especially as we use a small number of parameters. For each source model, domain adaptation was evaluated by transferring it to the two other target datasets. Twenty percent of the target cohort was used for few-shot fine-tuning, while the remaining 80% served as the held-out test set. Model performance was evaluated using AUC, accuracy, precision, recall, and F_1_-score before and after fine-tuning. Finally, model explainability was assessed using SHAP (SHapley Additive exPlanations) to compare feature contributions across domains.

Our transfer learning framework and research methodology are illustrated in Figure 1. All analyses were performed using Python (v3.12; Python Software Foundation, Wilmington, DE, USA). We assembled three T2DM cohorts from distinct geographic and socioeconomic contexts (Table 1). All of the datasets were available open-access, and each dataset had different variables with a few shared features (Table 1) [19,20,21]. For each cohort, we curated four routinely measured predictors, namely age, body mass index (BMI), mean arterial pressure (MAP), and blood glucose, alongside binary diabetic status (HbA1c ≥ 6.5% or documented diagnosis). MAP was computed in certain cohorts using available systolic (SBP) and diastolic (DBP) blood pressure values, with the following formula: MAP = DBP + 1/3(SBP − DBP). Continuous variables were standardized to have a mean of zero and a variance of one within each source cohort. Further details regarding preprocessing for each dataset are available in the Supplementary Material (Supplementary Text S1). To quantify the degree of domain shift across each cohort, pairwise H-divergences and Maximum Mean Discrepancy (MMD) were computed. The differences between cohorts were visualized by projecting each dataset onto a two-dimensional principal component space.

Our base learner was a lightweight multilayer perceptron comprising two hidden layers (32 and 16 units) with ReLU activations, culminating in a sigmoid-activated output node to estimate the probability of T2DM (Figure 1). Given the low dimensionality of the feature space, ReLU was preferred over other activation functions. We trained three “source” models independently (i.e., one per cohort) using binary cross-entropy loss optimized by Adam (learning rate 1 × 10^−3^, 20 epochs, batch size 32).

To assess domain adaptation, we performed six cross-cohort transfer experiments, pairing each source with each of the two other cohorts as target domains (e.g., a model trained on the Bangladesh source cohort was independently tested on the Iraq and Pima datasets). For each experiment, we held out one, five, ten, and twenty percent of the target data as a few-shot fine-tuning set and reserved the remaining for independent testing. Specifically, after pretraining on the source, we cloned its weights into a fresh network instance and then fine-tuned it on the two target domains.

For each experiment, we report the area under the receiver operating characteristic curve (AUC) and calibration performance using Brier score and expected calibration error (ECE) with threshold-agnostic predicted probabilities (fixed threshold = 0.5).

Finally, for direct clinical interpretation, we report a separate analysis at the 20% few-shot condition, where the classification threshold was tuned on the 20% fine-tuning subset with F1-optimization, and applied to the full target cohort to compare pre- and post-fine-tuning operating-point performance. To determine whether fine-tuning significantly changed the pattern of correct vs. incorrect predictions, we performed McNemar’s exact test on paired pre- vs. post-fine-tuning classifications, with a *p* < 0.05 considered significant. Model explainability was assessed using a kernel-based explainer, SHapley Additive exPlanations (SHAP); these were used on the fine-tuned models to compute bootstrapped SHAP values for the test-set observations and assess how drivers of predictions shifted across domains and after adaptation.

## 3. Results

Cross-cohort heterogeneity in demographic and metabolic profiles are tabulated in Table 2. The Bangladesh cohort was the youngest and leanest, with the lowest BMI and a relatively lower diabetes prevalence compared to the Iraq and Pima Indian cohort. The Iraq and Pima Indian cohort had participants with a markedly higher BMI, with the Pima Indian cohort having the greatest diabetes prevalence.

**Figure 2:**
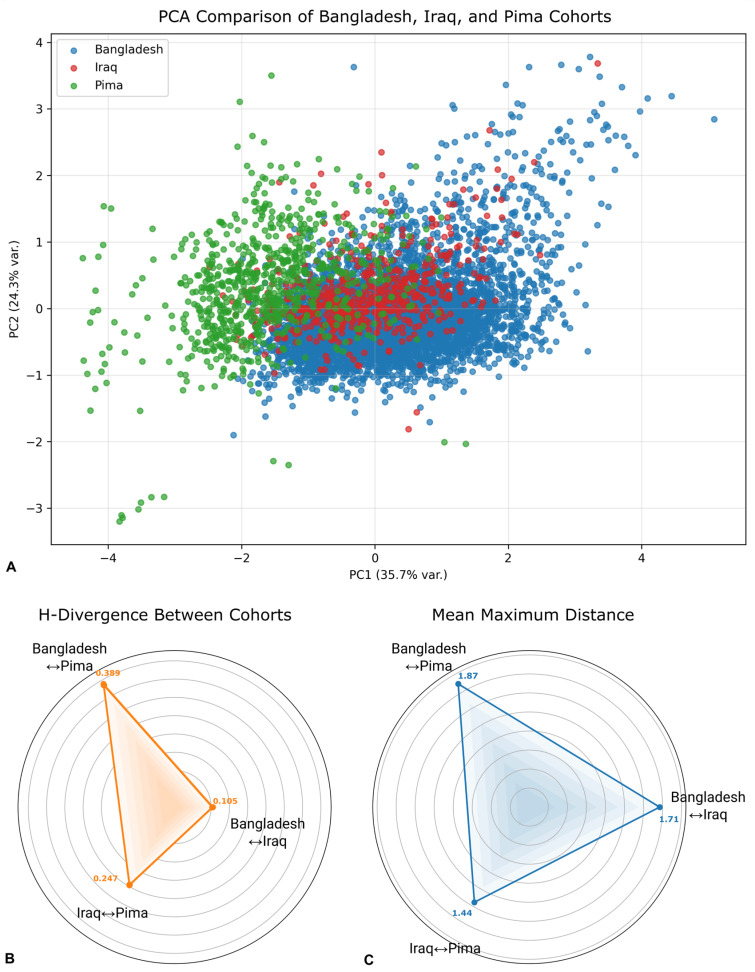
Domain Shift in Bangladesh, Iraq, and Pima Indian harmonized cohorts. (**A**) Two-dimensional principal component analysis (PCA) using harmonized features (Age, BMI, MAP, and Glucose) illustrates the distributional differences across the Bangladesh, Iraq, and Pima cohorts. (**B**) Pairwise H-divergences; larger values suggest greater domain shift. (**C**) Pairwise maximum mean discrepancy (MMD^2^) between cohort distributions; larger values suggest greater differences in distributions.

These differences were reflected in a Principal Component Analysis projection of the four harmonized features, where cohorts had formed partially overlapping but somewhat separable clusters (Figure 2). The pairwise domain-shift analysis (Figure 2B,C) demonstrates substantial differences in distributions across the cohorts, with the most significant shift occurring between Bangladesh and Pima (H-divergence = 1.87, MMD^2^ = 0.389). The most similar cohorts were Bangladesh and Iraq, although their divergences are still substantial.

**Figure 3:**
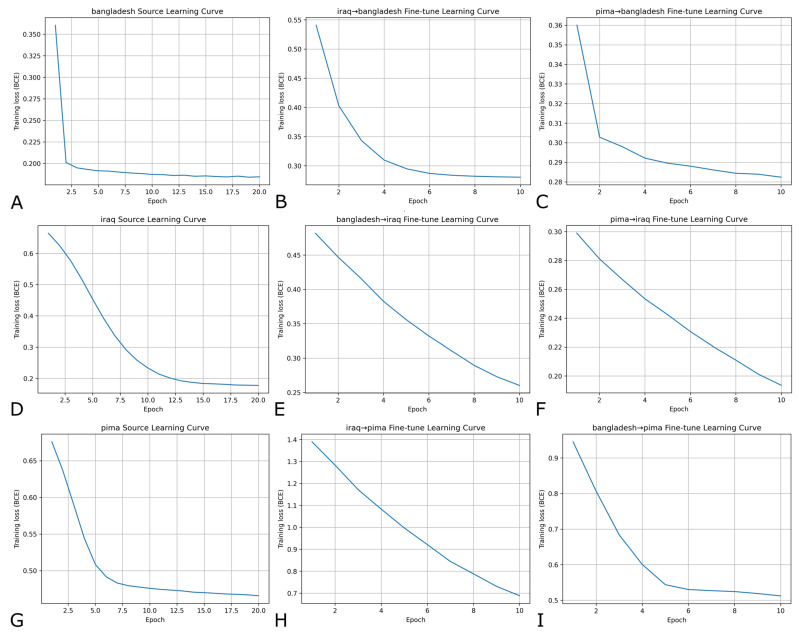
Source-domain and few-shot fine-tuning learning curves across all transfer directions. Panels (**A**,**D**,**G**) are the source-training learning curves for Bangladesh, Iraq, and Pima Indian cohorts, respectively. Panels (**B**,**C**,**E**,**F**,**H**,**I**) show their corresponding 10-epoch fine-tuning curves for each source-target transfer direction. These are for a 0.20 few-shot ratio with thresholding.

Source-learning had rapid convergence within the first few epochs (Figure 3). Fine-tuning models adapting to the same cohort followed a similar learning pattern, although in some cases, there were much shallower slopes; fine-tuning loss decreased steadily but did not always plateau within 10 epochs, suggesting incremental adaptation rather than overfitting.

**Figure 4:**
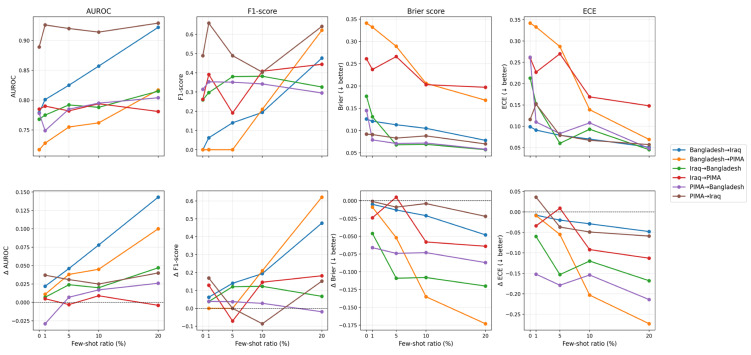
Few-shot domain adaptation performance across cross-population transfers. Top row shows absolute performance (AUROC, F1-score, Brier score, and ECE) at varying few-shot ratios. Bottom row shows their corresponding few-shot performance gains (Δ metrics) relative to the zero-shot baseline for each source → target direction. Lower Brier and ECE values suggest greater model calibration. ECE = Expected Calibration Error; AUROC = Area Under the Receiver Operating Characteristic curve.

Across all six source-target pairs, increasing the few-shot ratio generally improved cross-cohort transfer discrimination and calibration performance, with the largest gains appearing between 10% and 20% labeled target data (Figure 4; Supplementary Table S2). Even 1–5% few-shot samples were often enough to break “zero-recall” failures and boost F1: for example, Bangladesh → Iraq and Bangladesh → Pima both had F1 ≈ 0 at baseline, but few-shot fine-tuning at 1–5% target data increased recall from 0 to >3–7%. At the 1% few-shot ratio, transfer models trained on the Pima cohort were the most successful, with Pima → Iraq achieving a boost in F1 to 0.658 from 0.489, and Pima → Bangladesh reducing the Brier score by nearly 0.07, suggesting better probability calibration. Most other transfers had negligible changes at a 1% few-shot ratio.

For most few-shot ratios, improvements were most pronounced when transferring between clinically and demographically heterogeneous domains, such as Bangladesh and Iraq, where baseline cross-domain generalization was modest (AUROC 0.75–0.78; F1 often <0.30) but consistently rose with increasing few-shot exposure.

**Figure 5:**
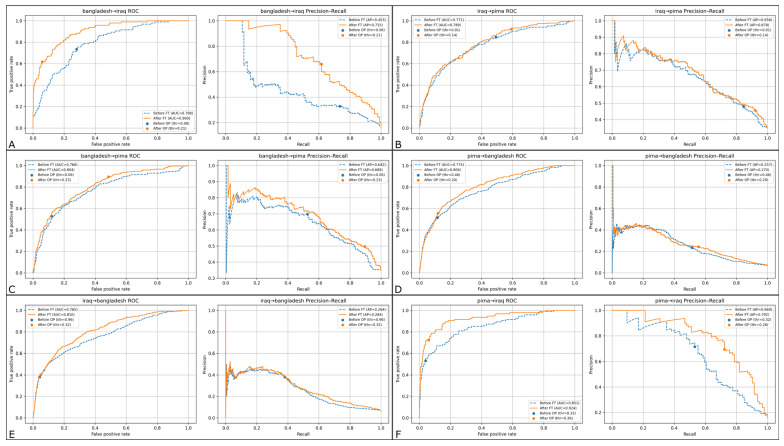
ROC and Precision–Recall curves for 20% few-shot domain adaptation with threshold tuning. Receiver Operating Characteristic (ROC) curves (**left** panels within each subgroup) and Precision–Recall (PR) curves (**right** panels) are shown for all six source → target transfer directions across the Bangladesh, Iraq, and PIMA cohorts. Each subplot compares the zero-shot baseline model to the 20% few-shot fine-tuned model, with decision thresholds optimized on the target support set. Few-shot adaptation consistently improves sensitivity–specificity tradeoffs and enhances precision–recall behavior, particularly in low-prevalence settings. Panels (**A**–**F**) correspond to Bangladesh → Iraq, Iraq → Bangladesh, Bangladesh → PIMA, PIMA → Bangladesh, Iraq → PIMA, and PIMA → Iraq, respectively.

Fine-tuning with 20% labeled target data produced the greatest and most consistent improvements in discrimination, precision-recall behavior, and calibration (Figure 5) with threshold-tuning. In all except one case (Pima → Bangladesh), AUROC increased after fine-tuning, with some of the largest gains in the most distributionally shifted directions. For example, Bangladesh → Iraq improved from AUROC 0.77 to 0.91 and F1 from 0.40 to 0.65; Iraq → Pima increased from AUROC 0.65 to 0.78 and F1 from 0.26 to 0.31. Improvements were also observed with calibration: for nearly all transfers, Brier scores dropped substantially (e.g., Bangladesh → Pima from 0.30 to 0.17; Pima → Bangladesh from 0.13 to 0.06; Iraq → Bangladesh from 0.18 to 0.06), and ECE decreased by 30–80%, suggesting that few-shot fine-tuning not only improved ranking performance but also yielded better calibrated probability estimates. McNemar’s test comparing pre- and post-fine-tuning predictions was statistically significant for all source–target pairs except Iraq → Bangladesh (*p* < 0.01 for all other directions); in other words, these gains reflected meaningful changes in classification decisions rather than random variation in thresholding.

**Figure 6:**
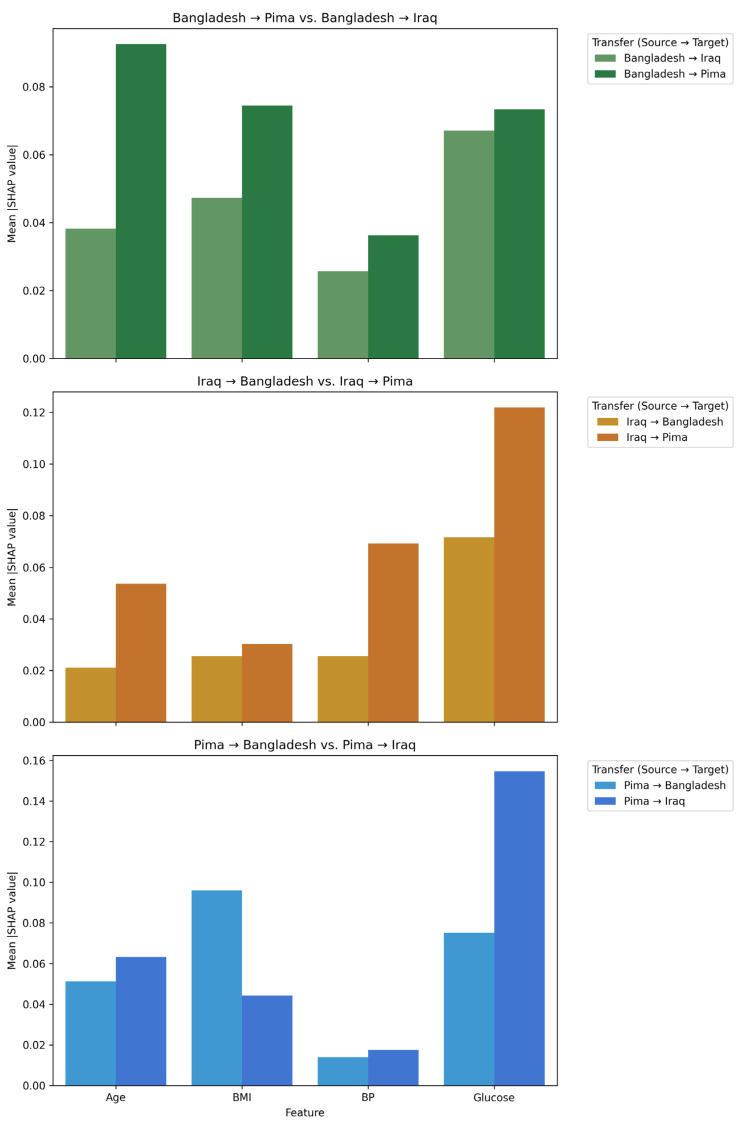
Mean SHAP values for models trained on Bangladesh (**top**), Iraq (**middle**), and Pima Indian (**bottom**) source datasets.

The mean absolute SHAP values for the four shared predictors are illustrated in Figure 6. Blood glucose was the strongest driver of predictions in nearly all settings, except for the Bangladesh → Pima and the Pima → Bangladesh domain transfers, where Age or BMI appeared to dominate instead, respectively. Blood pressure alone did not carry a strong influence on predictions except for the Iraq → Pima adaptation.

The global SHAP summaries can also be complemented by patient-specific explanations. For instance, in the Bangladesh → Iraq transfer, a patient with Age 61, BMI 28.2, MAP 93.3 mm Hg, and Glucose 159 was predicted to have diabetes with corresponding SHAP values of 0.046, 0.072, −0.028, and 0.046, respectively.

**Figure 7:**
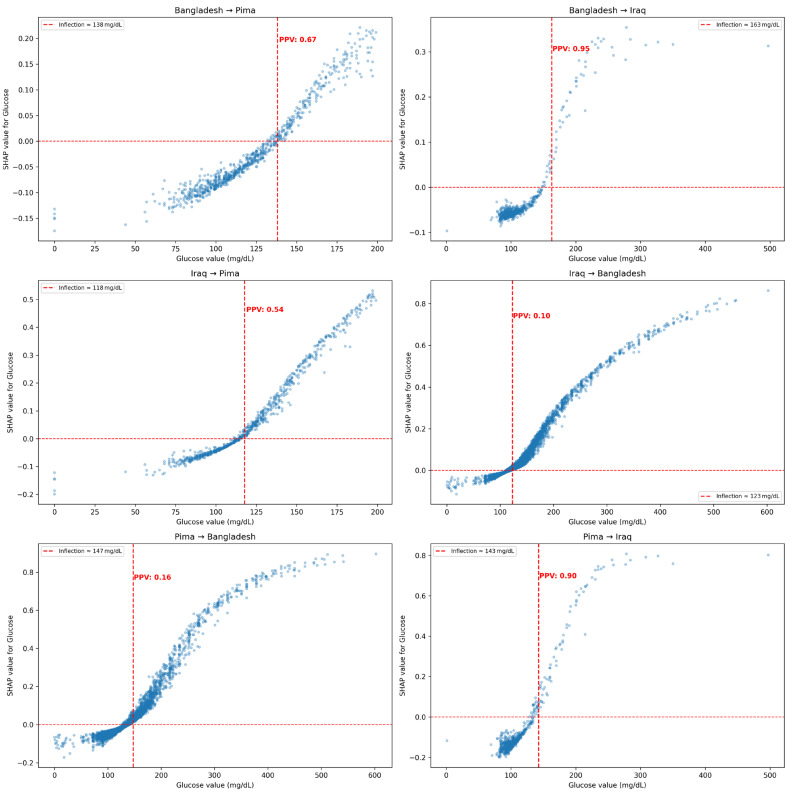
SHAP Value Dependency Plots for Blood Glucose Across Domain Transfers.

Bootstrapped SHAP bands for glucose and age are available in Supplementary Figures S1 and S2, respectively. The specific SHAP dependency relationships for blood glucose are depicted in Figure 7 across all six source → target transfers. A consistent nonlinear trend was present in each case, with minimal impacts from extremely low or high glucose concentrations; there were sharper SHAP changes around an inflection point specific to each cohort, reflecting the adapted model’s learned risk threshold. For instance, models generalizing to the Bangladesh cohort plot showed SHAP contributions climbing gradually after 110 mg/dL. In contrast, the models adapted to the Iraq dataset assigned positive risks sharply above 140–150 mg/dL. We estimated risk inflection points using the SHAP values, or the first glucose bin where the average SHAP value exceeds zero. To assess the clinical translation of these values, we also calculated the positive predictive value (PPV), defined as the proportion of patients with glucose values at or above the SHAP-based inflection who were truly diabetic in the target test set. Most transfers and their SHAP-based inflection points corresponded to PPV values from 0.54–0.95, except for all transfers to the Bangladesh cohort (PPV = 0.16).

Each scatterplot illustrates the relationship between raw blood glucose values and their corresponding SHAP values (i.e., contribution to diabetes risk prediction) for models fine-tuned via few-shot learning across six source → target cohort transfers. Positive Predictive Values for glucose thresholds determined by SHAP inflection points are provided.

## 4. Discussion

The aim of this analysis was to evaluate few-shot domain adaptation for T2DM risk prediction across three culturally and geographically distinct cohorts. Our transfer learning framework was limited to only four shared features: blood pressure, age, BMI, and blood glucose. After fine-tuning a simple MLP with just 20% of labeled data from the target domain, predictive performance across all transfer settings improved. Notably, models that initially failed to accurately identify true positives became clinically useful after fine-tuning, with marked gains in F1-score, recall, and, in some cases, AUC and accuracy. The SHAP-based interpretation also suggested that while blood glucose was an important predictor in most settings, the relative importance and threshold dynamics of features varied across populations.

The effectiveness of a screening test lies in its recall (sensitivity), whether it is based on a traditional scoring system or machine learning [22]. However, their reliability can vary when applied across different populations due to heterogeneity in data distributions, healthcare infrastructure, and measurement differences [23]. This aspect is known as domain shift, which can undermine the generalizability of models trained in one context when applied in another. The few-shot domain adaptation methodology we employed was similarly used in a study focused on diabetic retinopathy detection to improve the reliability and generalizability of screening tests [24]. They fine-tuned a pre-trained retinal image classifier on a few labels from new imaging devices or populations, finding significantly improved sensitivity and F1 scores. Although our study utilized tabular inputs, we also appreciated an enhanced precision-recall tradeoff, with improved F1 Scores.

Particularly, models trained exclusively on one cohort frequently failed to identify true positives when applied to another, as seen in the case of the Bangladesh → Iraq, Bangladesh → Pima, and Iraq → Pima experiments, likely due to shifts in feature distribution and different measurement conventions. By fine-tuning each source model on just a small percent of the labeled targets, the model sensitivities improved significantly, along with occasional gains in overall discrimination and calibration.

The SHAP-based analyses especially highlight how fine-tuning can reweigh predictors to align with each population’s epidemiological profile. Indeed, across all transfers, blood glucose remained the predominant driver of risk predictions; however, the inflection point, where SHAP values switch from a negative to positive risk propensity, shifted across cohorts. For example, models adapted to the Bangladesh cohort demonstrated a more gradual SHAP curve for blood glucose, with the risk inflection starting at earlier blood glucose concentrations at around 118 mg/dL. In contrast, models transferred to the Iraq cohort demonstrated sharp risk increases only beyond 150 mg/dL. Despite potential variability in post-prandial glucose sampling across the three cohorts, the SHAP-derived inflection points nonetheless were able to adapt. In several cases, the SHAP-based risk inflection points, when set as glucose thresholds, corresponded to a strong PPV. While these cohort-specific inflection points have not been assessed in other studies, it is agreed that the standard risk thresholds for diabetes are not necessarily universal [25]. For example, a study from the Hisayam cohort in Japan identified diagnostic thresholds for diabetes that were lower than global standards for both fasting glucose and HbA1c [26].

Transfer learning is not new in the context of diabetes and precision medicine. Deng et al. explored transfer learning to improve blood glucose prediction by adapting a general model to individual patients’ data [27]. Pre-training a model on a broader population and fine-tuning it on individual data improved glucose prediction accuracy compared to non-transfer baselines, a pattern similar to the one observed in our study. Whereas Deng et al. focus on time-series glucose predictions based on continuous glucose monitoring data, we explore cross-national, population-level transfer learning towards diabetes risk screening. To our knowledge, our study is the first to apply few-shot domain adaptation in a global health context, particularly for T2DM risk stratification in culturally distinct patients.

A benefit of few-shot transfer learning is its ability to enable model generalization across different contexts and feature representations, especially if the outcome is defined or measured inconsistently. For example, the task space for the Iraq dataset required binarizing HbA1c instead of relying on a pre-labeled diagnostic outcome. Similarly, while some cohorts reported glucose as fasting or random measurements, others provided glucose values without further specification. Transfer learning offers flexibility in aligning disparate yet related features. Another study focused on glucose forecasting across three diabetic datasets, each with varied measurement contexts [28]. Here, an adversarial multi-source transfer learning model was able to learn a shared, transferable feature representation, addressing the heterogeneity within the feature space.

Nevertheless, our study has some limitations. First, our feature space was restricted to only four clinical variables, which limits the complexity of our models. While parsimonious constraints allow for equitable comparisons across datasets, our analysis does not examine how other important metabolic and lifestyle predictors vary across different contexts and ultimately impact risk. Second, although we were able to improve transfer with up to 20% of the target data for few-shot fine-tuning, a cross-validation-based approach may be more suitable in other scenarios where subsets may not be fully representative; the current approach increases the risk of overfitting in few-shot fine-tuning. Finally, our evaluation was limited to retrospective datasets. Future studies can incorporate transfer learning in a prospective context to assess performance in real-world screening settings. Furthermore, inclusion of other lifestyle/exercise related features would allow for broader application of this technique toward healthcare maintenance globally.

## 5. Conclusions

T2DM remains a major global health concern, with rising incidence in both high-income and low- and middle-income countries. The goal of this study was to assess whether few-shot domain adaptation could improve the cross-population generalizability of diabetes risk prediction models trained on minimal, shared clinical features. With a simple multilayer perceptron and only four shared predictors, our transfer learning framework greatly improved performance across six different source-target domain parts, improving F1-scores and calibration even in scenarios where baseline models had initially failed.

SHAP-based explainability analysis revealed both consistent and population-specific risk feature contributions, with blood glucose generally being the strongest driver; however, the risk thresholds varied across populations.

## Figures and Tables

**Table 1:** Summary of dataset characteristics across the Bangladesh, Iraq, and Pima Indian cohorts, including sample sizes, data collection contexts, available clinical and demographic features, and outcome labeling. Asterisked (*) features indicate the subset of shared variables that are used in the transfer learning models. While the Bangladesh and Pima datasets label diabetes status as a binary classification, the Iraq cohort only provides HbA1c values. The time between the last meal and glucose measurement across datasets is not standardized.

Dataset	Size	Context	Feature Space	Task Space [20]
Bangladesh	5288	Community-based screening across 63 Unions in urban, semi-urban, and rural Bangladesh (2021–2024).	SBP *, DBP *, Age *, Sex, Glucose *, Height *, Weight *, Family History, hypertension, family history of hypertension, history of cardiovascular disease, stroke	Diabetic, Non-diabetic
Iraq	662	Data collected in private labs in Erbil from patients referred for diabetes testing by physicians.	SBP *, DBP *, Age *, BMI *, Sex, Random Blood Glucose *, Fasting Blood Glucose, Cholesterol, HDL, LDL, VDL, Family History, social life (urban/rural)	HbA1c
Pima Indians	768	Originally from the National Institute of Diabetes and Digestive and Kidney Diseases (NIDDK). Focused on adult Pima Indian women. Hosted by UCI ML Repository.	Pregnancies, Glucose *, MAP *, skin thickness, insulin, BMI *, Age *	Diabetic, Non-diabetic

**Table 2:** Cohort Characteristics for Bangladesh, Iraq, and Pima Indian datasets. Age, BMI, BP (MAP), and Glucose are represented as a mean (standard deviation). * represents sample after listwise deletion. BMI = Body Mass Index; BP = Blood Pressure; MAP = Mean Arterial Pressure.

Cohort	N	Age	BMI	BP (MAP)	Glucose	Diabetic?
Bangladesh	5288	45.75 (22.5)	22.47 (8.8)	99.49 (14.6)	136.15 (53.0)	6.47%
Iraq	596 *	49.05 (13.9)	31.03 (20.6)	93.96 (24.9)	113.14 (38.81)	15.77%
Pima Indians	768	33.24 (11.8)	31.99 (7.90)	69.11 (19.4)	120.89 (31.97)	34.90%

## Data Availability

No new data were created or analyzed in this study. Code is available on GitHub (accessed on 8 June 2025): https://github.com/shrinitbabel/diabetes_transfer_learning.

## Supplementary Materials

The following supporting information can be downloaded at: https://www.mdpi.com/article/10.3390/medicina62010007/s1, Supplementary Text S1: Preprocessing, Class Balance, and Missing Data Handling. Supplementary Figure S1. Bootstrapped SHAP Value Dependency Plots for Blood Glucose Across Domain Transfers with 95% confidence bands. Supplementary Figure S2. Bootstrapped SHAP Value Dependency Plots for Age Across Domain Transfers with 95% confidence bands. Supplementary Table S1. Sensitivity of Iraq diabetes labels to alternative HbA1c cutoffs. Supplementary Table S2. Few-shot domain adaptation performance across cross-population transfers with different Few-Shot Ratios.
