# Supplementary material for: Few-Shot Transfer Learning for Diabetes Risk Prediction Across Global Populations
Source: Medicina (Kaunas). 2025 Dec 19;62(1):7. doi: 10.3390/medicina62010007 (PMC12843261; doi:10.3390/medicina62010007)
Supplement: Supplementary file 1 [file medicina-62-00007-s001.zip › medicina-3992237-supplementary.pdf]

## Supplementary Text S1: Preprocessing, Class Balance, and Missing Data Handling

For cross-cohort transfer learning, we restricted all analyses to four predictors available in each dataset toward a cohort-specific binary diabetes label: Age, Body Mass Index (BMI) (kg/m$^2$), Blood Pressure as a Mean Arterial Pressure (MAP), and glucose (mg/dL). Other features present in the original datasets (e.g., lipid profile, smoking status, demographic variables) were excluded to maintain a consistent feature space across the Bangladesh, Iraq, and PIMA datasets.

### Cohort-Specific Preprocessing

*Bangladesh (n=5288)*

1. Blood pressure was derived as

$$BP = \frac{1}{3}SBP + \frac{2}{3}DBP$$

   to approximate MAP.
2. Glucose measured in mmol/L was converted to mg/dL (mg/dL = mmol/L × 18)
3. Diabetes status ("diabetic": Yes/No) was set as a binary target label.
4. This dataset contained no missing values in any of the features used.

*Iraq (n=662)*

1. Blood pressure entries containing text (e.g., "120/80") were parsed using

$$BP = \frac{1}{3}SBP + \frac{2}{3}DBP$$

   Non-parsable or empty entries were treated as missing.
2. Fasting blood sugar (FBS) and random blood sugar (RBS) were consolidated into a single Glucose variable; wherein if FBS values were missing but RBS was available, RBS was used. If both FBS and RBS were missing, the glucose value remained missing.
3. Diabetes status was defined using HbA1C >= 6.5%, with sensitivity analyses at 6.0%, 6.3%, 6.7%, and 7.0% (reported separately; see Supplementary Figure 1).
4. The core dataset (Age, BMI, BP, Glucose, HbA1c) contained some missingness before cleaning:

| **Feature** | **Missing, %** |
|---|---|
| Age | 0% |
| BMI | 2.87% (19) |
| BP | 8.01% (53) |
| Glucose* | 0% |
| HbA1c | 0% |

*FBS was missing in 96.53% (639). Following consolidation with RBS, blood glucose no longer had any missingness.

For features with missing values, we assessed whether the missingness mechanism was compatible with a Missingness Completely at Random (MCAR) assumption. For BMI and BP, we created a binary missingness indicator and assessed the distribution of other covariates between missing and non-missing groups. For continuous covariates (e.g., age, BMI, BP, glucose), we used Welch's two-sample t-tests; for categorical variables (sex, diabetes target variable), we used Chi-square tests of independence as a standard omnibus test for MCAR. Herein, if the probability of being missing is independent of all observed variables, then missingness is consistent with MCAR.

In the Iraq cohort, BMI missingness (2.87%) had no significant association with Age, BP, Glucose, Sex, or the Target variable (all $p > 0.05$), making MCAR plausible for BMI. However, BMI missingness (8.01%) was significantly associated with Age (younger patients; $p = 0.044$) and BMI (lower BMI; $p = 0.016$). Because Missingness At Random (MAR) was the most appropriate assumption (rather than MCAR), and because the fraction of missing BP/BMI rows was modest (=<10%), our primary analysis used listwise deletion after deterministic glucose consolidation (FBS and RBS).

To ensure that results were not sensitive to this choice, we performed a multiple-imputation sensitivity analysis using a multivariate Iterative Imputer (scikit-learn), where we jointly imputed age, BMI, BP, and Glucose under a MAR model. Here, we repeated all source→target transfer experiments and compared model performance to the main analysis that used listwise deletion. Across all transfers involving Iraq, either as a source or target, AUROC changed by at most 0.07 (max $|\Delta AUC| = 0.069$), while calibration metric remained stable (max $|\Delta Brier| = 0.013$; max $|\Delta ECE| = 0.019$).

*Pima Indians (n=768)*

1. Age, BMI, BP, and Glucose were extracted directly from the dataset.
2. The original "Outcome" field served as the binary diabetes target label.
3. This dataset contained no missing values in any column.

**Class Balance**

Diabetes prevalence varied substantially across cohorts:

| **Cohort** | Diabetic | Non-diabetic | Prevalence |
|---|---|---|---|
| Bangladesh | 342 | 4946 | 6.5% |
| Iraq | 108 | 554 | 16.3% |
| Pima Indians | 268 | 500 | 34.9% |

These variations in class balance motivated the use of threshold tuning in the final few-shot analysis and calibration metrics (Brier score, ECE). Oversampling, class weighting, or other advanced class-balancing techniques (SMOTE) were intentionally avoided to simulate the real-world domain shifts expected in cross-cohort transfer learning.

**Normalization**

All continuous predictors (Age, BMI, BP, Glucose) were standardized using

$$x' = \frac{x - \mu_{\text{source}}}{\sigma_{\text{source}}}$$

using StandardScaler to fit solely on the source cohort. This prevents statistical leakage from the target into the source as well. All source→target transfers operated on this identical scaling assumption; the same scaler was applied to the fine-tuning subset and the full target evaluation set.

## Supplementary Figure S1

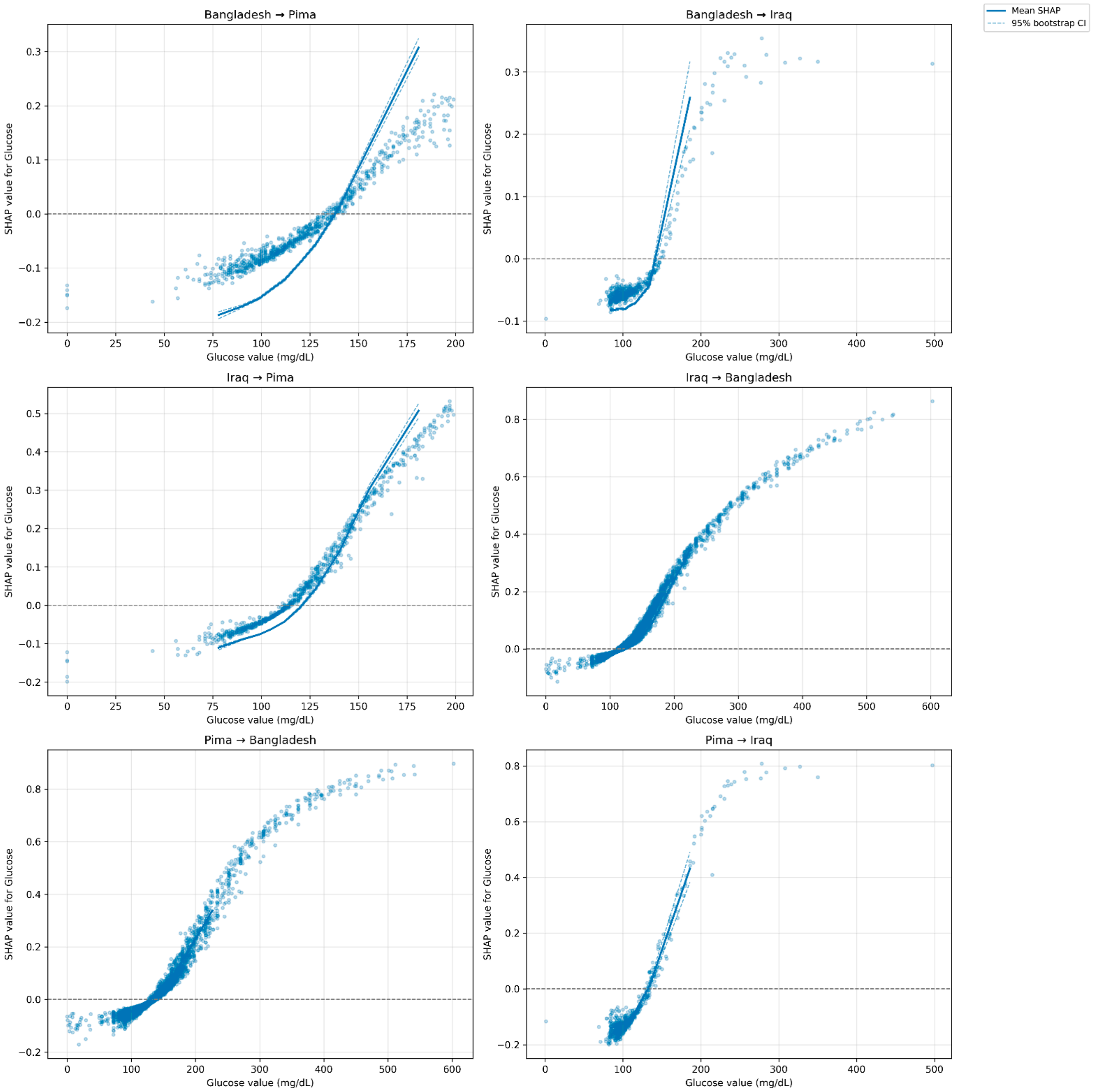


Supplementary Figure S1. Bootstrapped SHAP Value Dependency Plots for Blood Glucose Across Domain Transfers with 95% confidence bands.

## Supplementary Figure S2

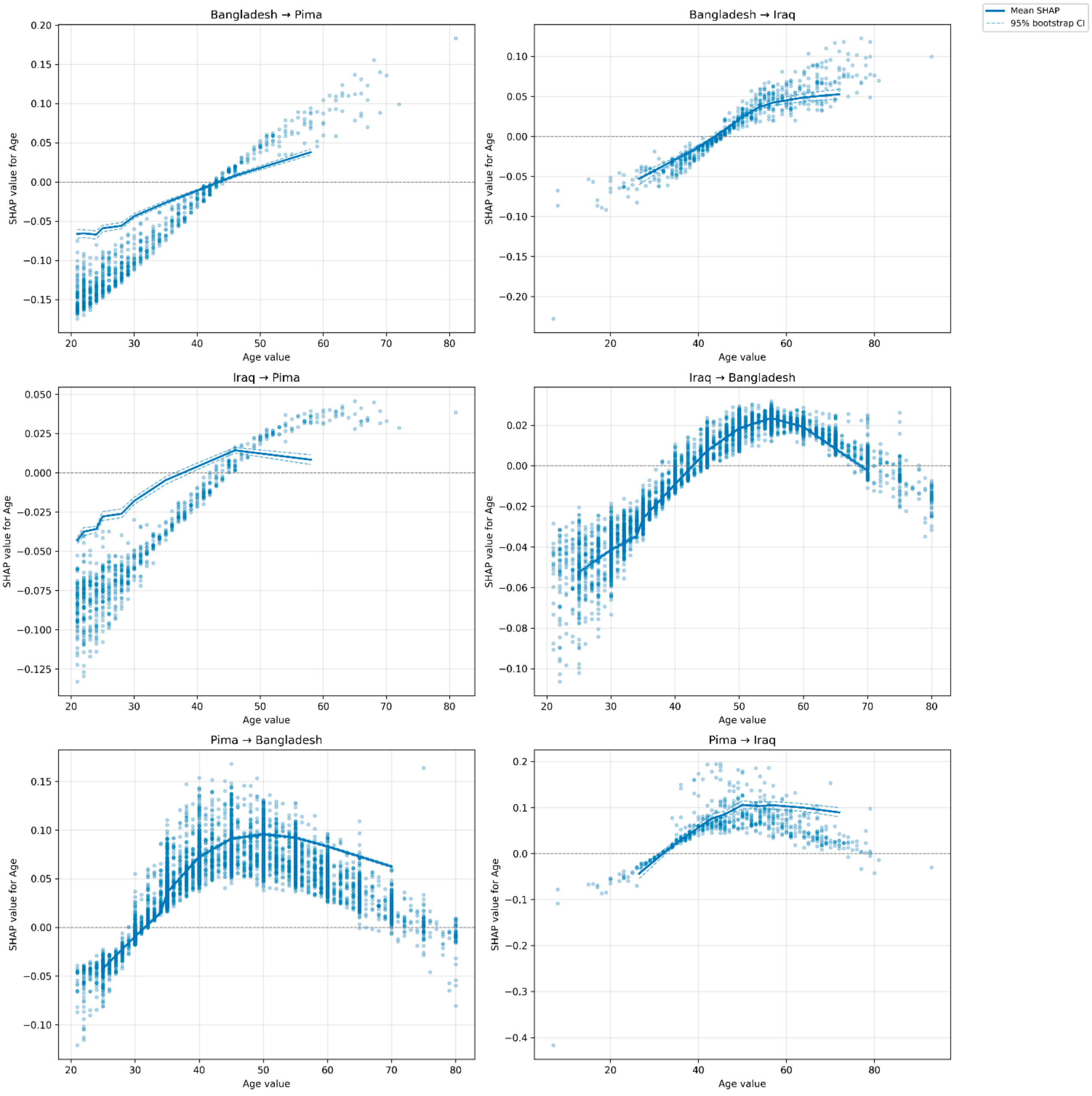


Supplementary Figure S2. Bootstrapped SHAP Value Dependency Plots for Age Across Domain Transfers with 95% confidence bands.

## Supplementary Table S1

| **HbA1c cutoff** | **N** | Diabetic, n (%) | Agreement with 6.5% | Reclassified from non-diabetic to diabetic (vs 6.5%) | Reclassified from diabetic to non-diabetic (vs 6.5%) |
|---|---|---|---|---|---|
| 6.0 | 662 | 167 (25.2%) | 0.911 | 59 | 0 |
| 6.3 | 662 | 128 (19.3%) | 0.970 | 20 | 0 |
| 6.5 | 662 | 108 (16.3%) | 1.000 | 0 | 0 |
| 6.7 | 662 | 92 (13.9%) | 0.976 | 0 | 16 |
| 7.0 | 662 | 76 (11.5%) | 0.952 | 0 | 32 |

Supplementary Table S1. Sensitivity of Iraq diabetes labels to alternative HbA1c cutoffs. Prevalence varies from 11.5% to 25.2%, but agreement with the 6.5% definition remains high (all >90%), with relatively few label flips; this suggests robustness of our results to the 6.5% HbA1c definition.

## Supplementary Table S2.

Few-shot domain adaptation performance across cross-population transfers with different Few-Shot Ratios.

| Source Country | Target | Few-Shot Ratio | Accuracy Original | Accuracy Few-Shot | AUC Original | AUC Few Shot | F1 Original | F1 Few Shot | Precision Original | Precision Few-Shot | Recall Original | Recall Few Shot | Brier Original | Brier Few Shot | ECE Original | ECE Few Shot | McNemar P |
|---|---|---|---|---|---|---|---|---|---|---|---|---|---|---|---|---|---|
| Bangladesh | Iraq | 0.01 | 0.843 | 0.848 | 0.779 | 0.801 | 0 | 0.062 | 0 | 1 | 0 | 0.032 | 0.126 | 0.121 | 0.099 | 0.091 | 0.25 |
| Bangladesh | Iraq | 0.05 | 0.843 | 0.855 | 0.779 | 0.825 | 0 | 0.14 | 0 | 1 | 0 | 0.075 | 0.126 | 0.113 | 0.099 | 0.079 | 0.0156 |
| Bangladesh | Iraq | 0.1 | 0.843 | 0.86 | 0.779 | 0.857 | 0 | 0.194 | 0 | 1 | 0 | 0.108 | 0.126 | 0.105 | 0.099 | 0.07 | 0.00195 |
| Bangladesh | Iraq | 0.2 | 0.85 | 0.889 | 0.818 | 0.922 | 0.082 | 0.476 | 1 | 0.909 | 0.043 | 0.323 | 0.119 | 0.078 | 0.091 | 0.051 | 1.52E-05 |
| Bangladesh | PIMA | 0.01 | 0.651 | 0.651 | 0.717 | 0.728 | 0 | 0 | 0 | 0 | 0 | 0 | 0.341 | 0.332 | 0.342 | 0.333 | nan |
| Bangladesh | PIMA | 0.05 | 0.651 | 0.651 | 0.736 | 0.755 | 0 | 0 | 0 | 0 | 0 | 0 | 0.335 | 0.289 | 0.336 | 0.287 | nan |
| Bangladesh | PIMA | 0.1 | 0.651 | 0.686 | 0.743 | 0.762 | 0 | 0.21 | 0 | 0.865 | 0 | 0.119 | 0.328 | 0.206 | 0.33 | 0.139 | 7.43E-06 |
| Bangladesh | PIMA | 0.2 | 0.652 | 0.764 | 0.773 | 0.817 | 0.007 | 0.621 | 1 | 0.708 | 0.004 | 0.552 | 0.292 | 0.168 | 0.293 | 0.069 | 2.25E-09 |
| Iraq | Bangladesh | 0.01 | 0.767 | 0.827 | 0.768 | 0.775 | 0.259 | 0.297 | 0.163 | 0.201 | 0.632 | 0.564 | 0.177 | 0.131 | 0.213 | 0.153 | 2.48E-74 |
| Iraq | Bangladesh | 0.05 | 0.767 | 0.917 | 0.768 | 0.792 | 0.259 | 0.38 | 0.163 | 0.368 | 0.632 | 0.392 | 0.177 | 0.068 | 0.213 | 0.06 | 4.75E-169 |
| Iraq | Bangladesh | 0.1 | 0.767 | 0.922 | 0.768 | 0.788 | 0.259 | 0.382 | 0.163 | 0.389 | 0.632 | 0.374 | 0.177 | 0.069 | 0.213 | 0.093 | 4.35E-172 |
| Iraq | Bangladesh | 0.2 | 0.748 | 0.932 | 0.766 | 0.815 | 0.247 | 0.326 | 0.153 | 0.453 | 0.637 | 0.254 | 0.187 | 0.057 | 0.227 | 0.045 | 2.07E-191 |
| Iraq | PIMA | 0.01 | 0.691 | 0.72 | 0.785 | 0.79 | 0.262 | 0.391 | 0.792 | 0.812 | 0.157 | 0.257 | 0.261 | 0.237 | 0.261 | 0.227 | 0.000113 |
| Iraq | PIMA | 0.05 | 0.655 | 0.681 | 0.769 | 0.782 | 0.029 | 0.191 | 0.8 | 0.829 | 0.015 | 0.108 | 0.307 | 0.266 | 0.314 | 0.27 | 0.000325 |
| Iraq | PIMA | 0.1 | 0.656 | 0.724 | 0.771 | 0.794 | 0.029 | 0.408 | 1 | 0.811 | 0.015 | 0.272 | 0.281 | 0.203 | 0.288 | 0.169 | 1.35E-08 |
| Iraq | PIMA | 0.2 | 0.651 | 0.729 | 0.743 | 0.781 | 0 | 0.444 | 0 | 0.783 | 0 | 0.31 | 0.317 | 0.197 | 0.322 | 0.148 | 3.79E-09 |
| PIMA | Bangladesh | 0.01 | 0.842 | 0.916 | 0.778 | 0.749 | 0.314 | 0.353 | 0.218 | 0.352 | 0.561 | 0.354 | 0.145 | 0.079 | 0.262 | 0.11 | 9.84E-72 |
| PIMA | Bangladesh | 0.05 | 0.842 | 0.92 | 0.778 | 0.785 | 0.314 | 0.351 | 0.218 | 0.367 | 0.561 | 0.336 | 0.145 | 0.071 | 0.262 | 0.083 | 2.78E-74 |
| PIMA | Bangladesh | 0.1 | 0.842 | 0.924 | 0.778 | 0.795 | 0.314 | 0.342 | 0.218 | 0.386 | 0.561 | 0.307 | 0.145 | 0.072 | 0.262 | 0.108 | 4.52E-76 |
| PIMA | Bangladesh | 0.2 | 0.88 | 0.93 | 0.777 | 0.804 | 0.341 | 0.295 | 0.264 | 0.428 | 0.48 | 0.225 | 0.112 | 0.058 | 0.204 | 0.048 | 2.88E-39 |
| PIMA | Iraq | 0.01 | 0.887 | 0.91 | 0.889 | 0.926 | 0.489 | 0.658 | 0.842 | 0.823 | 0.344 | 0.548 | 0.092 | 0.091 | 0.116 | 0.152 | 0.00661 |
| PIMA | Iraq | 0.05 | 0.882 | 0.887 | 0.903 | 0.92 | 0.453 | 0.489 | 0.829 | 0.842 | 0.312 | 0.344 | 0.09 | 0.083 | 0.086 | 0.079 | 0.25 |
| PIMA | Iraq | 0.1 | 0.877 | 0.875 | 0.892 | 0.914 | 0.416 | 0.403 | 0.812 | 0.806 | 0.28 | 0.269 | 0.097 | 0.088 | 0.116 | 0.067 | 1 |
| PIMA | Iraq | 0.2 | 0.868 | 0.907 | 0.878 | 0.929 | 0.304 | 0.641 | 0.895 | 0.817 | 0.183 | 0.527 | 0.092 | 0.07 | 0.072 | 0.057 | 0.000431 |
